# Effect of virtual care in type 2 diabetes management – a systematic umbrella review of systematic reviews and meta-analysis

**DOI:** 10.1186/s12913-025-12496-0

**Published:** 2025-03-06

**Authors:** Sumathy Ravi, Gideon Meyerowitz-Katz, Cassia Yung, Julie Ayre, Kirsten McCaffery, Glen Maberly, Carissa Bonner

**Affiliations:** 1 Western Sydney Diabetes, Integrated and Community Health, Western Sydney Local Health District, Blacktown Road, Blacktown, NSW 2148 Australia; 2 Present Address: Sydney Health Literacy Lab, Sydney School of Public Health, Faculty of Medicine and Health, The University of Sydney, Sydney, NSW Australia; 3 School of Health and Society, University of Wollongong, Wollongong, NSW Australia; 4 Menzies Centre for Health Policy and Economics, The University of Sydney, Sydney, NSW Australia

**Keywords:** Type 2 diabetes, Virtual care, Telemedicine, Telehealth, Videoconferencing, Umbrella review

## Abstract

**Background:**

Diabetes is an increasingly prevalent and costly chronic disease worldwide, and a large cause of unnecessary disease burden. To address the growing burden of diabetes, care models should support management of diabetes in primary care to reduce reliance on overstretched hospital-based specialists services. Virtual care presents an opportunity to provide diabetes care remotely, potentially enhancing the accessibility and efficiency of healthcare services. This review aimed to identify existing evidence on the effectiveness of virtual care on diabetes management, and the extent to which video components are included in the evidence base.

**Methods:**

The protocol was registered in PROSPERO (CRD42022366125). Systematic search of the databases PubMed, Embase, Medline, Scopus, CINAHL and Cochrane CENTRAL, were conducted for studies on telemedicine, telehealth, or virtual interventions for type 2 diabetes management published between January 2011 to March 2022. The primary outcome was HbA1c, and secondary outcomes were blood glucose control, Body Mass Index (BMI), taking the prescribed medications, and self-management behaviour. The results were reported following the Preferred Reporting Items for Systematic Reviews (PRISMA) checklist. Quality of each review was appraised using the Joanna Briggs Institute (JBI) Critical Appraisal Checklist for Systematic Reviews and Research Syntheses.

**Results:**

From 10,708 articles, 63 underwent full-text review. Thirty systematic reviews were included. Overall quality of the included reviews was high. Among the 30 systematic reviews, there was significant overlap of the primary studies, with 48% of them appearing in multiple reviews. Of the 30 reviews, 28 reported that virtual care improved HbA1c compared to usual care. Meta-analysis of 16 reviews revealed a mean difference of -0.37% (-0.41% to -0.32%), I^2^ of 77.1%. Significant non-clinical impacts were noted for BMI and secondary outcomes. Most reviews (25/30) included some studies with video components, however these studies did not disaggregate the impact of video from other aspects of complex interventions such as web-based and telephone support.

**Conclusions:**

This umbrella review strengthens the evidence that virtual care significantly improves clinical outcomes in people with type 2 diabetes, primarily affecting HbA1c. Fewer studies addressed other health outcomes such as BMI and taking medications. Effectiveness of virtual care varies by demographic and clinical characteristics, emphasising the need to tailor virtual care interventions to maximise impact. Future research could directly compare and identify the most effective virtual care strategies for different populations, including those with lower digital literacy.

**Supplementary Information:**

The online version contains supplementary material available at 10.1186/s12913-025-12496-0.

## Highlights

• Virtual care presents a growing opportunity to provide diabetes care remotely, potentially increasing the accessibility and efficiency of healthcare services.

• Virtual care can improve both clinical and behavioural outcomes in type 2 diabetes management, though the effect varies by people with type 2 diabetes demographics and clinical characteristics.

• While most reviews included some studies with video components, most RCT evidence did not disaggregate the impact of video from other aspects of their virtual care interventions.

• Future research could explore how recent advancements in video conferencing and other digital strategies could enhance virtual care effectiveness for populations with various levels of digital literacy.

**Supplementary Information:**

The online version contains supplementary material available at 10.1186/s12913-025-12496-0.

## Background

Diabetes is a highly prevalent health condition that makes a substantial contribution to burden of disease worldwide [1]. Guidelines recommend that people with type 2 diabetes see their health care team at least every 3 months to manage blood glucose levels, medications, and other aspects of ongoing self-management. Such consistent engagement between people with type 2 diabetes and healthcare professionals for ongoing support for diabetes management and self-care helps to prevent diabetes related complications and improves quality of life [2]. However, the reality of providing face-to-face diabetes management and education to people with type 2 diabetes has several barriers. Visiting a health care team can be costly and time-consuming for people with type 2 diabetes, with evidence suggesting most people with type 2 diabetes do not see their health professionals enough [3]. The quality of care is also compromised by time constraints and fragmented care [4].

Virtual Care has been rising worldwide due to its potential to improve health care access and clinical outcomes, as well its ability to be used to promote effective diabetes management and education [5]. As per the World Health Organisation definition, telehealth encompasses all forms of remote health care services and virtual care describes the remote delivery of clinical services [6]. Virtual Care (VC) models incorporate telehealth and virtual interactions between provider-patient and provider-provider by video, telephone, and secure messaging such as mobile and web-based applications [7]. Though face-to-face care will always have a role, virtual care has several advantages such as easier access, minimal wait times and no travel to clinics, therefore less expensive diabetes care [5]. With increased and widespread access to internet and smart devices, recent advances in diabetes technology, and rapid changes during the COVID pandemic, virtual care has become more feasible and scalable than ever before [8].

Previous research suggests that *components* of virtual care interventions lead to positive outcomes for people with type 2 diabetes such as improved blood glucose control and knowledge to manage their diabetes [9, 10]. However, with the recent rapid shift to virtual care, there is still limited evidence when it comes to the evaluation of current *integrated* virtual care interventions in diabetes management, including more recent video conferencing technology. In this study we aimed to pool the evidence from existing systematic reviews to report the effectiveness of virtual care interventions for type 2 diabetes management through a systematic review of reviews and meta-analysis, with a focus on reviews since 2010 when video call technology became more widely available [11].

## Methods

This systematic review of reviews and meta-analysis was conducted in accordance with the Preferred Reporting Items for Systematic Review and Meta-Analyses (PRISMA) 2020 statement [12]. The protocol was registered with the International Prospective Register of Systematic Reviews (PROSPERO CRD42022366125) in October 2022 [13].

### Data sources and search strategy

We conducted systematic searches across the six key scientific databases PubMed, Embase, Medline, Scopus, CINAHL and Cochrane CENTRAL to identify relevant studies. Publications published from January 2011 to March 2022 were searched due to the advancement in technology since 2010 [11] and to identify studies on interventions with video call feature in diabetes management. In PubMed, a base search string was developed from extensive piloting of combination of the keywords ‘diabetes’, ‘virtual’, ‘telehealth’, and ‘telemedicine’. To ensure consistency across other databases System Review Accelerator (SRA) polyglot was used to convert the PubMed base search string *(diabetes[Title/Abstract]) AND ((virtual[Title/Abstract]) OR (telehealth[Title/Abstract]) OR (telemedicine[Title/Abstract]))* to each of the other five database’s relevant search strings (Suplemental file S1). System Review Accelerator (SRA), an online software, is a word frequency analyser to help with search strategy development, a search translator to speed up translation of searches from PubMed/Ovid MEDLINE to other major databases. The terms such as “short messaging system (SMS)” and “mHealth” were not included as search terms to ensure that the review was feasible and focused on the most relevant evidences i.e. “virtual”, “telehealth” or “telemedicine”.

### Study selection

Studies were eligible if they evaluated virtual care interventions for adults with type 2 diabetes and reported on HbA1c and/or secondary outcomes (BMI, blood glucose control, medication adherence, self-management behaviour). We included systematic reviews, randomised controlled trials (RCT), qualitative studies that were peer reviewed and published in English. This paper reports on systematic reviews only. Virtual care was defined as interventions that allow a healthcare practitioner to provide personalised feedback to the patient about forwarded clinical data. For the purposes of this review, virtual care included video conferencing, telephone consultation, remote patient monitoring, computerised systems for information storage and exchange, website and mobile applications [14].

We excluded studies that were scoping reviews, narrative reviews, conference abstracts, articles without an available full text, interventions that included participants with gestational or type 1 diabetes only, interventions using only websites, mobile apps and automated short message services that did not involve clinician feedback. Search results from each of the databases were imported to Covidence (2022), which detected and removed duplicates automatically, and allowed real time screening of titles and abstracts, as well as finding full text for selected articles. Covidence is a web-based collaboration software platform that streamlines the production of systematic and other literature reviews [15]. Two independent investigators (SR and GMK) screened the titles and abstracts on Covidence according to the criteria described above to determine the eligibility for inclusion and compared the lists to resolve any disagreements. The two investigators (SR and GMK) then independently reviewed the full text of selected studies to establish a final list of studies. Any discrepancies were resolved by consulting with the third and fourth investigators (CB and JA). The final list of selected studies was exported along with the full text for data extraction.

### Data extraction

SR created a data extraction form using MS Excel for this review to capture all the relevant and specific data on the study design, type of diabetes, inclusion and exclusion criteria, sample characteristics, mean duration of diabetes, intervention type, technology platform, intervention team and duration, mean difference in HbA1c between control and intervention groups, and secondary outcomes. The extraction form was piloted by SR using two review articles. The pilot process and extracted information were discussed with CB and JA to reach an agreement on the form. Two investigators (SR and CY) then independently conducted data extraction from the final selected studies using the agreed-upon form. The extracted data were compared and any disagreements resolved through discussion.

### Quality appraisal of included reviews

Assessment of the methodological quality was completed by two reviewers (SR and GMK) independently on all selected articles included in the systematic review using the Joanna Briggs Institute’s (JBI) Critical Appraisal Checklist for Systematic Reviews and Research Synthesis [16]. The tool consists of 11 criteria scored as yes, no, unclear, or not applicable, to determine the extent to which the review had addressed the possibility of bias in its design, conduct and analysis. The scoring system that was used in a previously published systematic review was adopted [17], categorised a paper as "low quality" if its results were below 50%, "moderate quality" if they ranged between 50 and 69%, and "high quality" if the results were above 70%. Any disagreements were resolved through discussions and consensus with a third reviewer (CB or JA).

### Systematic review analysis

The primary outcome was measured in terms of change in HbA1c between baseline and post intervention. HbA1c is recognised as a valuable indicator of treatment effectiveness in people with type 2 diabetes [2], because it reflects average glycemia over several months and is strongly correlated with diabetes complications [2, 18, 19]. Secondary outcomes extracted from the reviews included clinical outcomes (e.g. blood glucose control, BMI) and behavioural outcomes (e.g. taking medications, physical activity).

### Meta-analysis

We included studies that reported mean difference and 95% Confidence Interval of HbA1c and BMI in our meta-analysis. Meta-analytic estimates were aggregated using a weighted mean with an inverse variance model with random effects using the metan command in Stata 15, producing a forest plot of results. Heterogeneity was assessed using the I^2^ statistic. We aggregated the mean change in HbA1c and BMI across studies for the primary model and did not perform any secondary models. Where meta-analyses included more than one mean estimate of the treatment effect, we entered these as separate estimates.

## Results

**Fig. 1:**
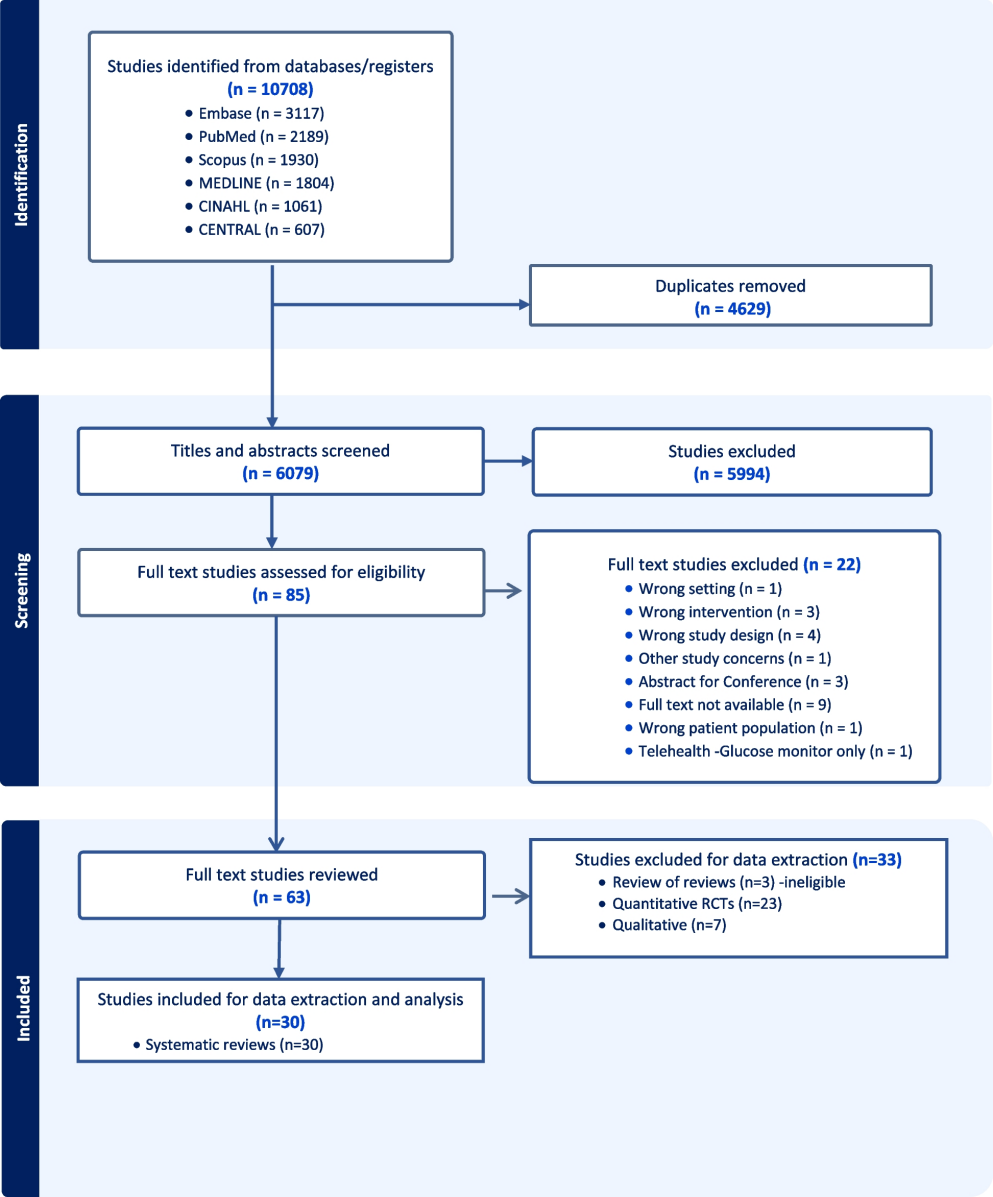
PRISMA flow chart of search strategy and study selection

Of the six databases searched, a total of 10,708 citations were identified and transferred to Covidence, which removed 4,629 duplicates. After title and abstract screening, a total of 85 studies were obtained for full text assessment. Of these studies, a further 22 studies were removed (see Fig. 1). The full text review resulted in a total of 63 studies that included 30 systematic reviews, 3 review of reviews, 23 RCTs, and 7 qualitative studies. This paper reports the findings of the 30 systematic reviews [20–49], as the 3 review of reviews did not cover all studies. An umbrella review of these systematic reviews and meta-analysis provided an opportunity to synthesise high-level evidence and identify the extent to which video components are included in the current evidence base.

### Characteristics of the included reviews (Table 1)

**Table 1:** Characteristics summary of all included reviews

Authors (Year)	Type of diabetes	No of studies	Primary outcomes (*n* = RCTs for meta-analysis)	Other outcomes	Video components
**Systematic Review and Meta-Analysis**					
**Anderson A (2022)** [47]	Type 2	10	Significant reduction in HbA1c MD − 0.465% (95% CI: − 0.648% to − 0.282%) *n* = 9	N/A	1 study conducted education sessions by interactive videoconferencing, which was effective in improving HbA1c
**Correia J C (2021)** [43]	Type 1, type 2 and gestational	31	Significant reduction of HbA1c MD −0.38% (95% CI: −0.52% to −0.23%) *n* = 28	Significant effect on fasting blood sugar, adherence to treatment, knowledge of diabetes and self-efficacy. No significant effect on BMI, total cholesterol, and triglycerides	4 studies identified videoconferencing as part of web-based systems, with mean difference of −0.89% (95% CI: −0.85 to 0.08)
**De Groot J (2021)** [44]	Type 2	43	Significant reduction in HbA1c MD −0.486% [95% CI −0.561% to −0.410%) *n* = 43	Significant reductions in blood pressure, blood glucose, weight. Improved mental and physical QoL	4 studies used interactive videoconference with mean reduction in HbA1c of 0.845% (95% CI −1.144 to −0.546, *P* < 0.001)
**Eberle C (2021)**^**[**^ [32]	Type 2	99	Significant reduction of HbA1c MD −1.15% (95% CI: − 1.84% to –0.45%) *n* = 2	Combination of real-time and asynchronous interventions most effective in improving fasting blood glucose, blood pressure, body weight, BMI, and quality of life	12 studies analysed videoconferencing and video consulting. 8 studies reported clear reduction in HbA1c values. Weekly video conferences showed significant decline is HbA1c and lower fasting blood glucose. No change in BMI, blood pressure and quality of life. In person visits showed significant weight loss
**Faruque L (2017)** [31]	Either type 1 or type 2 or both	111	Significant reduction of HbA1c MD—0.28%; 95% CI −0.37% to −0.20%) *n* = 87	No evidence of effect on QoL or mortality and reduced risk of hypoglycaemia	5 studies reported healthcare providers initiated communication using videoconferencing and 1 study involved video messaging. Videoconferencing with health care providers mainly included nurses and/or physicians. People with diabetes initiated communications did not involve any video components
**Hangaard S (2021)** [45]	Type 2	246	Significant reduction in HbA1c MD −0.415% [95% CI −0.482% to −0.348%) *n* = 168	No significant effect on BMI	Not included in search strategy and not reported in results
**Hu Y (2019)** [30]	Either type 1 or type 2 or both	14	Significant reduction of HbA1c MD −0.28% (95% CI: −0.45% to −0.12%) *n* = 13	No significant effect on BMI. MD −0.27; (95% CI:−0.86% to −0.31%); *n* = 7	Videoconferencing and video recording were part of search terms. Findings not reported
**Huang Z (2015)** [33]	Type 2	18	Significant reduction of HbA1c MD −0.54% (95% CI: −0.75% to −0.34%) *n* = 18	No significant differences in BMI, body weight, and hypoglycaemic events	Not reported as part of the interventions or results
**Lee S W H (2017)** [20]	Type 2	107	Significant reduction of HbA1c MD −0.43% (95% CI: −0.64% to −0.21%) *n* = 93	No significant changes in cardiovascular risk factors, risk of hypoglycaemia and quality of life	16 studies with telemonitoring and 11 studies with telementoring interventions, some of which included video conferencing
**Marcolino M S (2013)** [37]	Type 1 or type 2	15	Significant reduction of HbA1c MD −0.44% (95% CI: −0.61% to −0.26%) *n* = 13	Reduction in LDL-cholesterol, no effect on blood pressure and tendency to reduce BMI	1 study used videoconferencing as part of telemedicine strategies
**Michaud T L (2021)** [24]	Type 2	17	Significant reduction of HbA1c MD −0.30% (95% CI: −0.31% to −0.29%) *n* = 15	Weight change (kg)—MD of pre-post difference between telehealth and usual care was −0.62 (95% CI: −0.7 to −0.45)	2 studies had videoconferencing as part of real-time feedback and reported improvement in HbA1c
**Robson N (2021)** [48]	Type 2	29	Significant reduction of HbA1c MD −0.18% (95% CI: 0.35% to −0.01%) *n* = 21	Significant reduction in outpatient, Emergency Department (ED) visits, planned hospitalisations, and LDL cholesterol levels	3 studies used video teleconferencing and 1 study found statistically significant reduction in mean HbA1c levels. Other studies reported reduction in BP. 1 study used video education and reported significant reduction in mean HbA1c and significant improvement in body weight, BMI, waist circumference and fasting blood glucose
**So C F (2018)** [42]	Either type 1 or type 2	7	Significant reduction of HbA1c MD—0.64% (95% CI: −1.01% to −0.26%) *n* = 8	No significant effect on fasting plasma glucose (MD = −0.26%; 95% CI: −1.05% to 0.53%) *n* = 4	Not reported as part of the search or telehealth interventions
**Su D (2015)** [28]	Either type 1 or type 2 or both	92	Significant reduction of HbA1c post intervention varied from −3.2% to 0.70%. Mean ending HbA1c ranged from 6.26% to 9.21%; *n* = 92	No significant effect of having a nutritional counselling as part of the telemedicine interventions	11 studies used teleconference and 2 studies used educational videos. Nutritional counselling via SMS, telephone or videoconference showed similar effects
**Su D (2016)** [29]	Either type 1 or type 2 or both	49	Significant reduction of HbA1c pre and post-tests varied from 2.2% to 0.5% in intervention groups and from 1.3% to 0.6% in control groups; *n* = 55	Most effective for type 2 diabetes than type 1 and among people with diabetes of ages 40 or older. Interventions of 6 months or less showed greater effect than longer programs	2 studies used videoconferencing and 1 study included video messages. Effect of video conferencing not reported
**Tchero H (2019)** [21]	Type 1 and type 2	38	Significant reduction of HbA1c MD −0.37 (95% CI: −0.43% to −0.31%); *n* = 42	Most effective for type 2 diabetes than type 1 diabetes; among people with diabetes of ages 40 or older than younger population; and longer duration interventions of over 6 months	2 studies included interventions using live videoconferencing for remote consultations. 1 study used video messages. Effect of video conferencing not reported
**Wu C (2018)** [35]	Type 1 or type 2	19	Significant reduction of HbA1c MD −0.22% (95% CI: −0.28% to −0.15%) *n* = 16	Reduction of blood pressure. No change in BMI and quality of life	2 studies delivered education via videoconferencing. Effect of video conferencing not reported
**Zhai Y K (2014)** [25]	Type 2	47	Significant reduction of HbA1c MD −0.37 (95% CI: −0.49% to −0.25%); *n* = 35	2 studies analysed cost-effectiveness and revealed ICERs (Incremental cost-effectiveness ration) of $491 and $29,869 per capita for each unit reduction in HbA1c, for the telephone- and internet-based (includes video conferencing) interventions, respectively	1 study included live videoconferencing as part of IDEATel internet-based trial. Meta-analysis showed HbA1c reduction of 0.29% (95% CI: 0.12% to 0.46%)
**Systematic Review**					
**Cassimatis M (2012)** [23]	Either type 1 or type 2 or both	13	Significant improvement in HbA1c	5 out of 8 studies reported significant improvements in dietary adherence and physical activity. 4 out of 9 found significant improvement in blood glucose self-management frequency. 8 studies assessed medication adherence and only 3 reported improvements	1 study found ‘Persistent viewers’, who viewed more than 10 self-care video messages per month experienced a significant reduction in HbA1c of 0.6% over 12 months
**Greenwood D A (2014)** [38]	Type 2	15	Significant reduction in HbA1c levels	Not all 7 key elements of structured monitoring recommended by the IDF were included in telehealth remote patient monitoring interventions	3 studies used provider feedback methods by videoconferencing. 1 study used educational nuggets. Effect of videoconferencing was reported as part of the interventions
**Hossain M M (2019)** [27]	NCDs including diabetes	13	Significant reduction in HbA1c	Improvement in medication adherence, self-management, and lifestyle modification	1 study provided access to educational videos to the telephonic follow up intervention group. Significant reduction of HbA1c reported
**Jalil S (2015)** [39]	Type 2	19	Mixed results on the effectiveness of telemedicine on medical outcomes	Positive effect on behavioural improvements	1 study used video instructions. Effect not reported
**Kaveh M H (2021)** [22]	Either type 1 or type 2 or both	18	Biomedical outcomes: positive effect on glycaemic control; diet and exercise monitoring led to reducing BMI; improved cholesterol and blood pressure	Behavioural outcomes showed self-monitoring and self-efficacy; Psychosocial outcomes improved depression and increased social support. Quality of life improved	8 studies used videoconference interventions. Effective in improving behavioural outcomes
**Marsh Z (2021)** [26]	Either type 1 or type 2 or both	9	Significant reduction in HbA1c of 0.8%—1.3%	Enhanced diabetes self-management adherence and increased satisfaction of people with diabetes and HCPs	4 studies used videoconferencing. Effective in improving HbA1c and enhances communication between people with diabetes and healthcare professionals
**McDaniel C (2021)** [46]	Type 1, type 2, or prediabetes	21	Motivational Interviewing (MI) based telehealth seems most effective for improving A1C, systolic blood pressure, diabetes self-efficacy, and physical activity behaviours	Significant effect on behaviour change, medications, physical activities, and diabetes knowledge	1 study solely used videophone calls for nurse practitioner delivered motivational interviews. Identified as a research gap to evaluate the effect
**McLendon S F (2017)** [34]	Both type 1 and type 2	14	Significant reduction of (baseline 8.6 ± 0.3% telemedicine vs. 8.9 ± 0.4 usual care; completion 6.6 ± 0.2 telemedicine vs. 8.1 ± 0.2% usual care, *P* = 0.02)	Improved patient empowerment, self-care, adherence to diet, glucose monitoring, access to specialist care and preference to use technology	All studies included video consultation. Improved glycaemic control after one video consultation and continued to show progress in lowering HbA1c levels
**Mushcab H (2015)** [40]	Type 2	19	Real-time management and remote monitoring of T2DM people with type 2 diabetes resulted in a significant decrease in HbA1c level	mixed results for clinical outcome measures such as HbA1c, BMI, and cholesterol level. High acceptance of technology	Studies involving videoconferencing interventions were excluded
**Sim R (2021)** [49]	Type 2	20	Higher patient satisfaction could be achieved by understanding patient preferences and technology support	Support for technology adoption. Increases Access and saves time	2 studies used videoconferencing as part of the interventions- Enablers and barriers identified
**Van Den Berg N (2012)** [36]	Cardiovascular and diabetes	68 (18 diabetes)	Improved glycaemic control for people with diabetes with mean age between 60 and 69 years	Clear trend towards better results for behavioural outcomes, such as adherence to medication or diet, physical activity, self-efficacy, and managing the disease compared to medical outcome-categories	19 studies delivered telemedicine case management included videoconferencing. Effective in improving personal contact and better outcomes
**Wickramasinghe S I (2016)** [41]	Diabetes	14	N/A	Enablers and barriers to telemedicine identified	1 study used videoconferencing for specialist’s consultations. Effective in increasing number of teleconsultations

Studies are sorted as Systematic reviews with meta-analysis and Systematic reviews; and Authors in alphabetical order

The search strategy identified 30 systematic reviews, of which 18 included a meta-analysis, and one included a network meta-analysis. Included systematic reviews were published between 2012 and 2022, with the majority (*n* = 24; 80%) reporting fewer than 50 studies, 3 articles between 50–100 and 3 articles with more than 100 studies. The number of participants ranged from 10 to 23,648 in individual studies. Of the 30 reviews, 13 focused exclusively on populations with type 2 diabetes, while 14 included mixed populations, consisting of individuals with type 2 diabetes, and/or type 1, gestational diabetes, and pre-diabetes. Four out of 14 reviews reported findings separately for people with type 2 diabetes. Three reviews included diabetes and other conditions but did not specify the type of diabetes. The reviews covered 68 countries in 6 continents.

Virtual care interventions included either stand-alone interventions or combinations of teleconsultation, tele-education, tele-case management, device-based telemonitoring, tele-mentoring, mobile health, video messages, automated text-messages, video conferencing, real-time data transmission and feedback, eye screening, telemetry, digital decision aid, virtual counselling, telecare, secure messaging within a patient portal, diabetes diary app, mobile app and web-based virtual care with blood glucose and pressure devices. Twenty five out of 30 reviews included a minority of studies with video components, such as video consultation, video conferencing or video messages as part of the virtual care interventions.

### Clinical outcomes

**Fig. 2:**
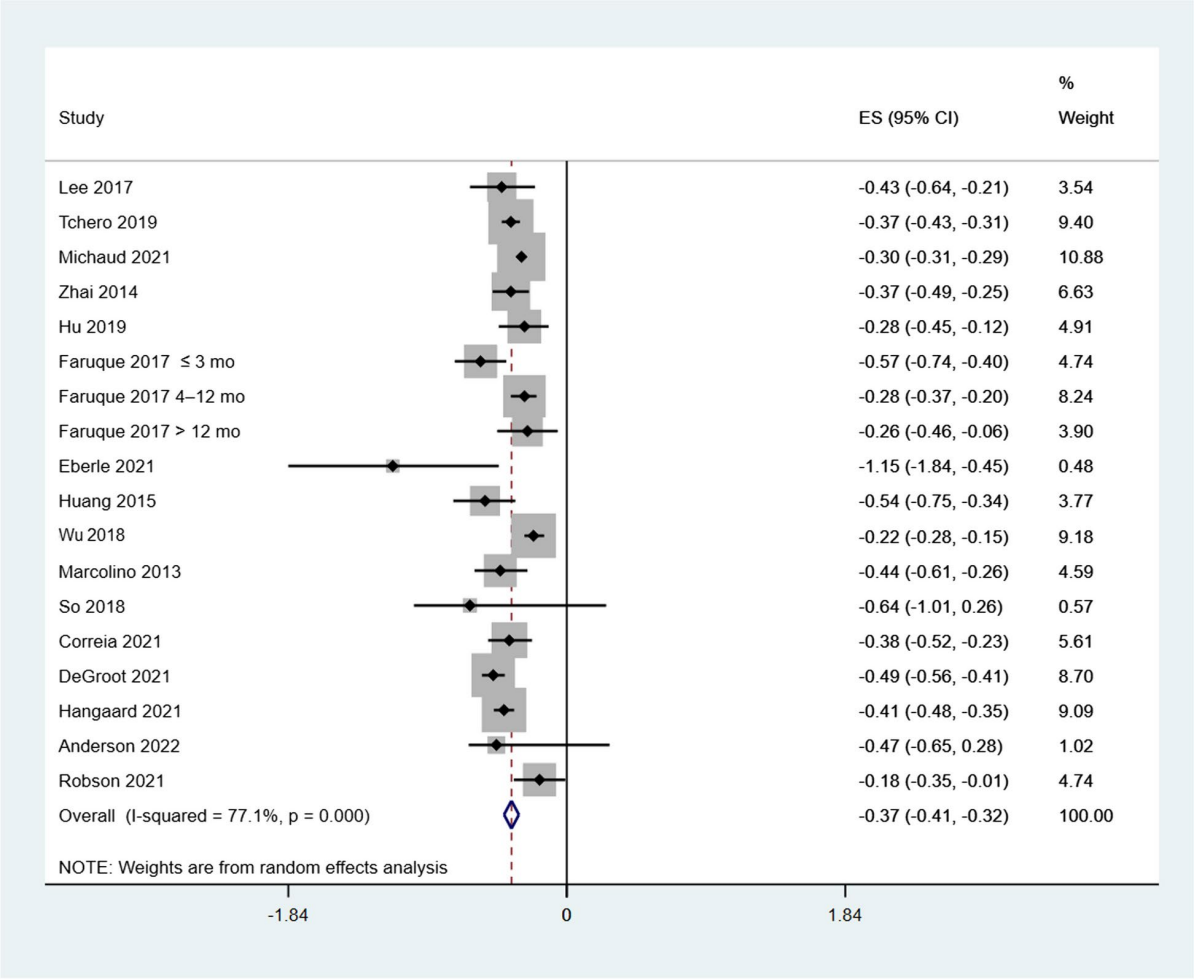
Forest plot of the meta-analysis of HbA1c of the included reviews

Almost all (28/30) reviews analysed HbA1c as their primary outcome and all reported significant reduction of HbA1c for people with diabetes. About half (16/30) of the systematic reviews covering 681 unique trials were included in our meta-analytic estimate (Fig. 2). The mean reduction in HbA1c was 0.37% (95% CI: −0.41% to −0.32%). Heterogeneity was high, with an I^2^ of 77.1%, likely reflecting differences in type of telehealth interventions, baseline HbA1c levels, duration of follow up, and educational content included in these studies. One review, Michaud 2021 [24], contributed an unexpectedly high weighting on the initial analysis because of its narrow CI bounds. This review was then excluded for a sensitivity analysis. Exclusion of this review did not significantly alter the results or heterogeneity [MD −0.38% (95% CI: −0.44 to 0.32, I^2^ = 71.8)]. (Supplemental File S2).

**Fig. 3:**
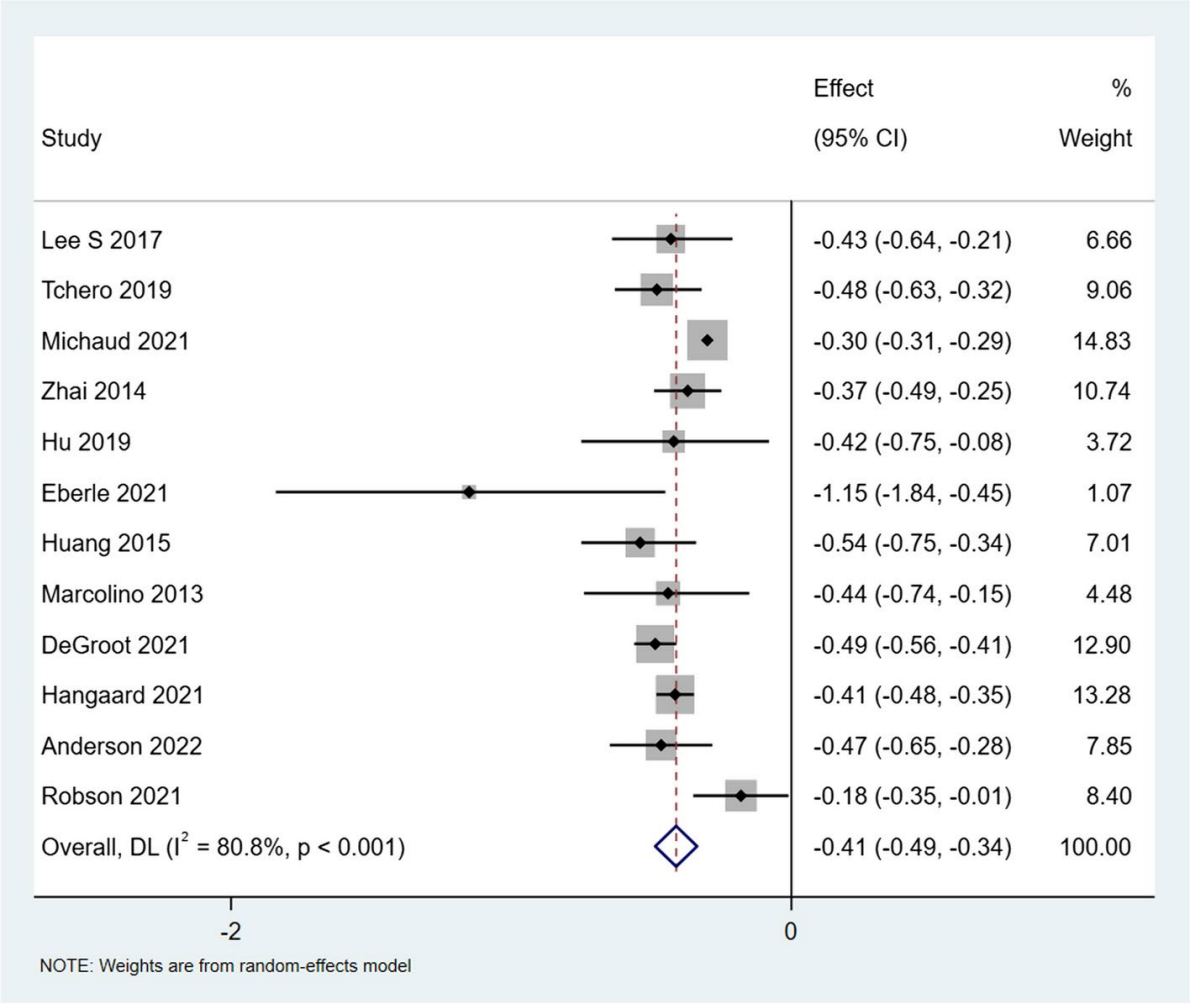
Forest plot of the meta-analysis of HbA1c of the included reviews with type 2 diabetes

Further, to specifically explore the effect of virtual care on HbA1c levels for type 2 diabetes, four reviews [31, 35, 42, 43] that did not provide disaggregated outcomes for type 2 diabetes were excluded for a sensitivity analysis. Figure 3 presents the sensitivity analysis of the sub-sample of 12 remaining studies with only HbA1c measures for people with type 2 diabetes. Findings from this analysis shows a mean reduction in HbA1c of 0.41% (95% CI: −0.49 to 0.34; I^2^ = 80.8%) which is similar in direction and effect size for all diabetes populations.

**Fig. 4:**
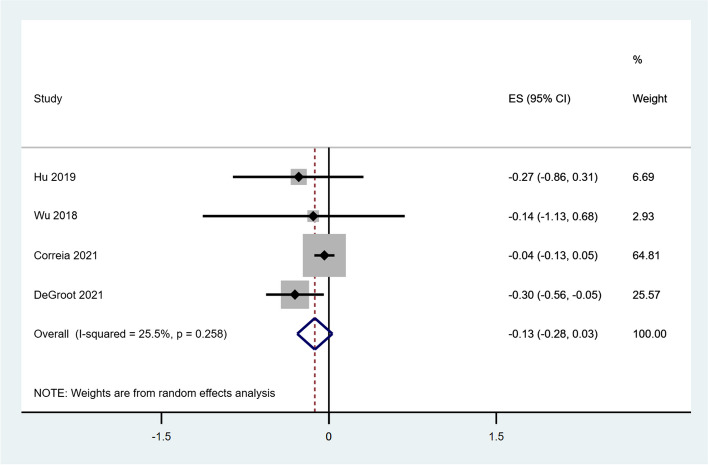
Forest plot of the meta-analysis of BMI

Eight out of thirty reviews described the effect of virtual care on BMI as a secondary outcome measure. Four reviews were included for meta-analyses and there was no statistically significant difference of BMI between virtual care interventions and usual care, with only a reduction of 0.13 [95% CI: −0.28 to 0.03; I2 = 25.5%] (Fig. 4). Only one study [44] included in this analysis reported the BMI measure for type 2 diabetes and all other three reviews included combined results for mixed type of diabetes.

Seven reviews analysed the effect of virtual care on fasting blood glucose and blood pressure, out of which 2 reviews reported reduction in fasting blood glucose [32, 46] and all 7 reviews [22, 27, 32, 35, 37, 44, 46] reported reduction in blood pressure. Six reviews reported non-clinically significant improvements to lipid profile and four reviews showed reduction in body weight or improved weight loss, but not statistically significant.

### Behavioural outcomes

A total of 12 reviews analysed and reported a positive tendency on behavioural outcomes, whilst 2 reviews reported that virtual care improved medication adherence [27, 36]. Similarly 3 reviews looked at the impact on diet [23, 35, 36] and physical activity [23, 35, 46] and all reviews reported that virtual care had a tendency to improve these and other behavioural outcomes.

### Quality of life

Of the 8 reviews that assessed the effect of virtual care on quality of life [20, 22, 31, 32, 34, 35, 41, 44], 5 reported on cost and access barriers to health care. Six reviews reported the effect on self-management or care [22, 23, 26, 27, 35, 48], and 6 reported on self-efficacy [22, 27, 35, 36, 43, 46]. Seven out of these 8 reviews reported a positive effect in improving these outcomes.

### Effectiveness of diverse types of virtual care

Two reviews compared different virtual care strategies and found that no single strategy was superior for reducing HbA1c [20, 48]. Of the 3 reviews that analysed the effect of teleconsultation interventions vs usual care, all reported significant effects on lowering HbA1c levels [20, 21, 29]. Five reviews evaluated telemonitoring compared to usual care and all of them reported that telemonitoring was effective. Of those, 3 reviews reported telemonitoring combined with tele-education [44] and telemonitoring combined with healthcare professional feedback [45, 48] had greater effects. While four reviews [26, 27, 37, 43] assessed if virtual care was effective in facilitating feedback and interactions between people with diabetes and healthcare professionals, one review [26] reported virtual care was effective in enhancing patient-provider interactions. A further 3 reviews reported that individualised feedback, either through automated algorithms or health care professionals, improved HbA1c control [24, 38, 48]. There were 5 reviews evaluating virtual care components; 3 reviews [28, 31, 43] reported that interventions as simple as text-messaging or SMS feedback were equally effective in improving HbA1c, compared to a teleconference or telephone appointment with healthcare professionals.

### Patient characteristics

Virtual care interventions were found to have greater effect among adults who were 50 years or older, with better treatment outcomes compared to younger adults reported in 5 reviews [21, 22, 26, 29, 36]. Virtual care increased older adults’ adherence to self-care activities such as blood glucose Level (BGL) checks, diet, exercise, foot and eye checks [26].

### Timeframe

Intervention duration was analysed in three reviews [29, 35, 46]. These reviews suggest that virtual care programs of at least 6 months were more effective in terms of reducing HbA1c levels compared to programs shorter than 6 months or longer than one year. Interventions between 6 and 12 month were described as more effective because the benefits of virtual care may occur in the first few months or become more difficult to implement robustly and keep people with type 2 diabetes engaged over the longer term [37]. One review suggested that any evaluation less than 3 months would not be sufficient because of the time required to educate people with type 2 diabetes with virtual care systems or devices [30].

### Video components

The majority (25 out of 30 reviews) described studies with a video component such as video consultation, video conferencing or video messages as part of the virtual care interventions and results. However, many studies included in the reviews did not specify the isolated effect of a video component on the outcomes. Three reviews [32, 34, 44] reported that video conferencing was effective in improving HbA1c levels over periods ranging from 3–12 months. Specifically, more frequent interaction by weekly video conferencing showed greater results in reducing HbA1c levels. One review concluded that interactive video telehealth technologies might be effective in enhancing access to quality care, contribute to patient empowerment and self-care management [34]. One review reported that short video messages on self-education topics sent to mobile phones every 24 h were effective, as participants who viewed 10 or more videos showed 0.6% reduction in HbA1c [23].

### Cost-effectiveness

Among 3 reviews [25, 32, 34], 2 have indicated a positive effect of cost savings due to less travel, wait times and increased access to services. Cost-effectiveness outcomes compared markedly different approaches, revealing a wide variation in costs associated with virtual care [26]. Real-time feedback from health care professionals via video conferencing may be costly compared to automated telehealth interventions [24]. In addition, teleconsultation (non-video remote consultations) was found to be more cost-effective than remote monitoring due to various devices and data usage costs [29]. The use of SMS text messaging could be an effective and cost-efficient way to communicate and motivate people with type 2 diabetes, potentially leading to positive outcomes. Messages generated using automated algorithms may offer a more feasible and less expensive solution [31].

### Quality assessment

Findings from the JBI Critical Appraisal showed that 22 reviews (73.3%) assessed the methodological quality of included reviews. Of those, 12 (40.0%) reported that at least two reviewers performed the appraisal. Around half of the reviews (*n* = 14, 46.6%) used the Cochrane Collaboration tool to assess the quality of studies included in the review. One review used a modified tool to do so, and 3 reviews used non-validated tools. Among the 30 systematic reviews assessed, 18 (60%) were deemed high quality with ‘YES’ for 8 to 11 assessment criteria. Of these, 4 reviews met all 11 criteria, and the remaining 14 reviews reported quality issues due to appropriate methods, substantial risk of bias owing to the nature of the interventions, reported allocation concealment and blinded assessment of outcomes. The remaining 12 reviews included 7 deemed as moderate quality and 5 to be of low quality. The appraisal results are provided in Supplemental file S3.

## Discussion

### Principal findings

Overall, this comprehensive analysis of 30 systematic reviews and meta-analyses suggests that virtual care can be effective in enhancing clinical and behavioural outcomes in people with type 2 diabetes. Our meta-analysis showed that virtual care is associated with a statistically significant reduction of 0.37% (0.41% for type 2 diabetes) mean HbA1c compared with standard care in randomised clinical trials. For people with type 2 diabetes, reducing the mean HbA1c level by 1% would be related to a 21% reduction in diabetes-related death and a 37% reduction in microvascular complications, such as neuropathy, retinopathy, and blindness [19].

Results of our study also indicated that virtual care improved behavioural outcomes, including improvements in treatment and medication adherence, self-efficacy, and quality of life. Virtual care may be particularly effective for older adults, especially those aged 50 and above [23, 29], and for people with type 2 diabetes with higher baseline HbA1c levels [33]. Consistent with previous findings, virtual care showed mixed effects on BMI, fasting blood glucose, lipids, blood pressure, and body weight, but these were not statistically or clinically significant. The extracted results for these outcomes were limited and heterogeneous, making it difficult to draw definitive conclusions about the effectiveness of virtual care on these parameters [40, 45].

Findings from this umbrella review align with recent evidence that virtual care interventions, whether short term or long term, are clearly effective in improving the management of type 2 diabetes [48]. All the reviews included a wide range of virtual care interventions, often combining different approaches to support clinical management, such as personalised interactions or feedback between providers and individuals with diabetes. Since all the interventions incorporated management and/or communication systems as integral components of virtual care, it was not feasible to align within a specific framework based on intended purpose and eHealth classification [50]. Our review also highlights the lack of trials that evaluated interventions that use virtual care to provide comprehensive and *integrated* diabetes management, rather than isolated components. Further research is needed to understand which components of virtual care are essential for improving health outcomes, including the role of newer video technologies. While interventions and outcome measures related to remote monitoring, self-management, and educational aspects are frequently evaluated, it remains challenging to draw solid conclusions about the feasibility or compliance with specific types of virtual care.

### Comparison with previous research

Our study includes 15 systematic reviews that were not included in 3 previous umbrella reviews, including 11 systematic reviews published in the emergency phase of the COVID-19 pandemic, which accelerated virtual care due to lockdowns. One recent review [51] analysed the effect of telemedicine across all health conditions, with very few studies focusing on diabetes. Findings from the review suggested that telemedicine has no effect on clinical outcomes. Our current study shows that whilst virtual care may not be universally beneficial for health outcomes, it does seem to play a specific and significant role in the context of diabetes. The findings suggest that virtual care is effective in reducing HbA1c levels if it includes blood glucose monitoring and feedback from healthcare professionals. This is consistent with previous research on telehealth remote monitoring using Self-Monitoring of Blood Glucose (SMBG) transmission devices [52, 53]. Our findings from this review suggest that virtual care had a greater impact on older people with type 2 diabetes over 50 years of age in managing their diabetes effectively, which contradicts another umbrella review that reported virtual care had a greater effect on younger people with type 2 diabetes aged 41 to 50 years [53]. Further investigations are needed to develop evidence if this effect between different age group populations relates to digital health literacy and adoption of technology.

## Limitations

In this review robust methods were used to generate high-quality evidence on the effects of virtual care in diabetes management. Although a comprehensive and piloted search strategy was developed using the broad set of keywords, it is possible that some relevant studies would have been missed. As we aimed to capture complex interventions with video components, which did not align well with established search terms, MeSH terms were, therefore not used to avoid missing relevant articles. In addition, we found significant overlap among the primary studies of the included meta-analyses of 847 studies, out of which 404 were at least duplicates. This 47.7% overlap affects the accuracy of our meta-analysis estimates, as it represents a significant portion of the research would have been duplicated in our meta-analysis. The main method of statistically eliminating this bias would be to recalculate each study’s estimates excluding duplicated studies within. Given the enormous number of meta-analyses reviewed in this project, we considered such an analysis was not feasible for the scope of this study. Therefore, we included this overlap number as a potential limitation that may have impacted the estimates, but we have not mitigated it as such in our analysis. In addition, it is possible that reporting bias within the included reviews could have impacted which studies were included, which may have also biased our findings. This limitation applies across the literature and is a fundamental problem with meta-syntheses of this type. Another limitation of this review is that some of the included studies did not provide disaggregated outcomes for type 2 diabetes. As a result, the findings may be biased, as they have pooled data from mixed type of diabetes populations, potentially obscuring the true effects specifically for individuals with type 2 diabetes.

## Future research

While meta-analytic results favour virtual care over standard care in improving HbA1c levels, we do not know which specific strategies underpin these benefits. This review highlights the need for more research on the long-term impact, equity, and cost-effectiveness of comprehensive virtual care models, including video conferencing and telehealth devices [54]. There is also a need to identify which components are most effective for populations with lower digital literacy. Current research inadequately reports on providers and people with type 2 diabetes’ satisfaction and adoption of virtual care technologies. In addition, further studies are needed to evaluate implementation barriers. Providers may feel overwhelmed by technological advancements, data management, and navigating systems during virtual visits [55]. People with type 2 diabetes may also exhibit varying preferences and levels of skills with different technologies, especially video features. Adoption of technology is more likely when systems address patient-identified clinical and behavioural priorities [56]. Understanding and tailoring for these diverse needs and preferences is a crucial step towards virtual care models and strategies that offer appropriate and effective care.

## Conclusion

This umbrella review shows that virtual care has significant potential to improve outcomes in people with type 2 diabetes. Evidence from systematic reviews and meta-analyses demonstrates the effectiveness of virtual care interventions for clinical outcomes, with mixed evidence for behavioural outcomes. Videoconferencing provides a valuable platform for real-time interaction and feedback between people with type 2 diabetes and healthcare providers. The effectiveness of virtual care interventions varies based on demographic and clinical characteristics, highlighting the importance of customising these interventions to maximise their impact. Future research should prioritise integrated virtual care models to enhance effectiveness across outcomes, and adaption for different populations.

## Supplementary Information

Supplementary Material 1.

Supplementary Material 2.

Supplementary Material 3.

## Data Availability

Data is provided within the manuscript and the supplemental information files.

## References

[1] International Diabetes Federation. IDF Diabetes Atlas, 10th edn. Brussels, Belgium: 2021. Available at: https://www.diabetesatlas.org.

[2] American Diabetes Association Professional Practice C. 6. Glycemic Goals and Hypoglycemia: Standards of Care in Diabetes-2024. Diabetes Care. 2024;47(Suppl 1):S111-S25. 10.2337/dc24-S006.1/16/2024. 10.2337/dc24-S006 PMC10725808 38078586

[3] Nam S, Chesla C, Stotts NA, Kroon L, Janson SL. Barriers to diabetes management: patient and provider factors. Diabetes Res Clin Pract. 2011;93(1):1–9. 10.1016/j.diabres.2011.02.002. 21382643 10.1016/j.diabres.2011.02.002

[4] Manski-Nankervis JA, Furler J, Blackberry I, Young D, O’Neal D, Patterson E. Roles and relationships between health professionals involved in insulin initiation for people with type 2 diabetes in the general practice setting: a qualitative study drawing on relational coordination theory. BMC Fam Pract. 2014;15(1):20. 10.1186/1471-2296-15-20. 24479762 10.1186/1471-2296-15-20 PMC3909758

[5] Iyengar V, Wolf A, Brown A, Close K. Challenges in Diabetes Care: Can Digital Health Help Address Them? Clin Diabetes. 2016;34(3):133–41. 10.2337/diaclin.34.3.133.1/9/2024. 27621530 10.2337/diaclin.34.3.133 PMC5019009

[6] World Health Organisation. Telemedicine: Opportunities and Developments in Member States, in Global Observatory for Ehealth Series. WHO: Geneva, Switzerland. 2009:p. 93.

[7] Dinesen B, Nonnecke B, Lindeman D, Toft E, Kidholm K, Jethwani K, et al. Personalized Telehealth in the Future: A Global Research Agenda. J Med Internet Res. 2016;18(3):e53. 10.2196/jmir.5257. 26932229 10.2196/jmir.5257 PMC4795318

[8] Smith AC, Thomas E, Snoswell CL, Haydon H, Mehrotra A, Clemensen J, et al. Telehealth for global emergencies: Implications for coronavirus disease 2019 (COVID-19). J Telemed Telecare. 2020;26(5):309–13. 10.1177/1357633X20916567. 32196391 10.1177/1357633X20916567 PMC7140977

[9] Esmatjes E, Jansa M, Roca D, Perez-Ferre N, del Valle L, Martinez-Hervas S, et al. The efficiency of telemedicine to optimize metabolic control in patients with type 1 diabetes mellitus: Telemed study. Diabetes Technol Ther. 2014;16(7):435–41. 10.1089/dia.2013.0313. 24528195 10.1089/dia.2013.0313

[10] Gonzalez-Molero I, Dominguez-Lopez M, Guerrero M, Carreira M, Caballero F, Rubio-Martin E, et al. Use of telemedicine in subjects with type 1 diabetes equipped with an insulin pump and real-time continuous glucose monitoring. J Telemed Telecare. 2012;18(6):328–32. 10.1258/jtt.2012.120103. 22912487 10.1258/jtt.2012.120103

[11] Chawla A. Coronavirus (COVID-19)– ‘Zoom’ Application Boon or Bane SSRN. 2020. 10.2139/ssrn.3606716.

[12] Page MJ, McKenzie JE, Bossuyt PM, Boutron I, Hoffmann TC, Mulrow CD, et al. The PRISMA 2020 statement: an updated guideline for reporting systematic reviews. BMJ. 2021;372:n71. 10.1136/bmj.n71. 33782057 10.1136/bmj.n71 PMC8005924

[13] Ravi S, Meyerowitz-Katz G, Ayre J, McCaffery K, Maberly G, Bonner C. Virtual Care for type 2 diabetes: definitions, implementation and effectiveness compared to usual care. PROSPERO 2022 CRD42022366125. 2022. Available from: https://www.crd.york.ac.uk/prospero/display_record.php?ID=CRD42022366125.

[14] ISO 13131 - Health informatics - Telehealth services - Quality planning guidelines ISO. Available from: https://www.iso.org/standard/75962.html.

[15] Covidence systematic review software, Veritas Health Innovation, Melbourne, Australia. Available at www.covidence.org.

[16] Aromataris E, Fernandez R, Godfrey CM, Holly C, Khalil H, Tungpunkom P. Summarizing systematic reviews: methodological development, conduct and reporting of an umbrella review approach. Int J Evid Based Healthc. 2015;13(3):132–40. 10.1097/XEB.0000000000000055. 26360830 10.1097/XEB.0000000000000055

[17] Samadbeik M, Staib A, Boyle J, Khanna S, Bosley E, Bodnar D, et al. Patient flow in emergency departments: a comprehensive umbrella review of solutions and challenges across the health system. BMC Health Serv Res. 2024;24(1):274. 10.1186/s12913-024-10725-6. 38443894 10.1186/s12913-024-10725-6 PMC10913567

[18] Nathan DM, Genuth S, Lachin J, Cleary P, et al. The effect of intensive treatment of diabetes on the development and progression of long-term complications in insulin-dependent diabetes mellitus. N Engl J Med. 1993;329(14):977–86. 10.1056/NEJM199309303291401. 8366922 10.1056/NEJM199309303291401

[19] Stratton IM, Adler AI, Neil HA, Matthews DR, Manley SE, Cull CA, et al. Association of glycaemia with macrovascular and microvascular complications of type 2 diabetes (UKPDS 35): prospective observational study. BMJ. 2000;321(7258):405–12. 10.1136/bmj.321.7258.405. 10938048 10.1136/bmj.321.7258.405 PMC27454

[20] Lee SWH, Chan CKY, Chua SS, Chaiyakunapruk N. Comparative effectiveness of telemedicine strategies on type 2 diabetes management: A systematic review and network meta-analysis. Sci Rep. 2017;7(1):12680. 10.1038/s41598-017-12987-z. 28978949 10.1038/s41598-017-12987-z PMC5627243

[21] Tchero H, Kangambega P, Briatte C, Brunet-Houdard S, Retali GR, Rusch E. Clinical Effectiveness of Telemedicine in Diabetes Mellitus: A Meta-Analysis of 42 Randomized Controlled Trials. Telemed J E Health. 2019;25(7):569–83. 10.1089/tmj.2018.0128. 30124394 10.1089/tmj.2018.0128

[22] Kaveh MH, Faradonbeh MR, Kaveh S. Telehealth impact on biomedical, psychosocial, and behavioural outcomes in patients with diabetes older than 50 years: A systematic synthesis without meta-analysis. J Telemed Telecare. 2024;30(2):285–304. 10.1177/1357633X211052222. 34792400 10.1177/1357633X211052222

[23] Cassimatis M, Kavanagh DJ. Effects of type 2 diabetes behavioural telehealth interventions on glycaemic control and adherence: a systematic review. J Telemed Telecare. 2012;18(8):447–50. 10.1258/jtt.2012.gth105. 23209266 10.1258/jtt.2012.gth105

[24] Michaud TL, Ern J, Scoggins D, Su D. Assessing the Impact of Telemonitoring-Facilitated Lifestyle Modifications on Diabetes Outcomes: A Systematic Review and Meta-Analysis. Telemed J E Health. 2021;27(2):124–36. 10.1089/tmj.2019.0319. 32397845 10.1089/tmj.2019.0319

[25] Zhai YK, Zhu WJ, Cai YL, Sun DX, Zhao J. Clinical- and cost-effectiveness of telemedicine in type 2 diabetes mellitus: a systematic review and meta-analysis. Medicine (Baltimore). 2014;93(28):e312. 10.1097/MD.0000000000000312. 25526482 10.1097/MD.0000000000000312 PMC4603080

[26] Marsh Z, Nguyen Y, Teegala Y, Cotter VT. Diabetes management among underserved older adults through telemedicine and community health workers. J Am Assoc Nurse Pract. 2021;34(1):26–31. 10.1097/JXX.0000000000000595. 33859074 10.1097/JXX.0000000000000595

[27] Hossain MM, Tasnim S, Sharma R, Sultana A, Shaik AF, Faizah F, et al. Digital interventions for people living with non-communicable diseases in India: A systematic review of intervention studies and recommendations for future research and development. Digit Health. 2019;5:2055207619896153. 10.1177/2055207619896153. 31897307 10.1177/2055207619896153 PMC6920343

[28] Su D, McBride C, Zhou J, Kelley MS. Does nutritional counseling in telemedicine improve treatment outcomes for diabetes? A systematic review and meta-analysis of results from 92 studies. J Telemed Telecare. 2016;22(6):333–47. 10.1177/1357633X15608297. 26442959 10.1177/1357633X15608297

[29] Su D, Zhou J, Kelley MS, Michaud TL, Siahpush M, Kim J, et al. Does telemedicine improve treatment outcomes for diabetes? A meta-analysis of results from 55 randomized controlled trials. Diabetes Res Clin Pract. 2016;116:136–48. 10.1016/j.diabres.2016.04.019. 27321329 10.1016/j.diabres.2016.04.019

[30] Hu Y, Wen X, Wang F, Yang D, Liu S, Li P, et al. Effect of telemedicine intervention on hypoglycaemia in diabetes patients: A systematic review and meta-analysis of randomised controlled trials. J Telemed Telecare. 2019;25(7):402–13. 10.1177/1357633X18776823. 29909748 10.1177/1357633X18776823

[31] Faruque LI, Wiebe N, Ehteshami-Afshar A, Liu Y, Dianati-Maleki N, Hemmelgarn BR, et al. Effect of telemedicine on glycated hemoglobin in diabetes: a systematic review and meta-analysis of randomized trials. CMAJ. 2017;189(9):E341–64. 10.1503/cmaj.150885. 27799615 10.1503/cmaj.150885 PMC5334006

[32] Eberle C, Stichling S. Effect of Telemetric Interventions on Glycated Hemoglobin A1c and Management of Type 2 Diabetes Mellitus: Systematic Meta-Review. J Med Internet Res. 2021;23(2):e23252. 10.2196/23252. 33595447 10.2196/23252 PMC7929744

[33] Huang Z, Tao H, Meng Q, Jing L. Management of endocrine disease. Effects of telecare intervention on glycemic control in type 2 diabetes: a systematic review and meta-analysis of randomized controlled trials. Eur J Endocrinol. 2015;172(3):R93-101. 10.1530/EJE-14-0441. 25227131 10.1530/EJE-14-0441

[34] McLendon SF. Interactive Video Telehealth Models to Improve Access to Diabetes Specialty Care and Education in the Rural Setting: A Systematic Review. Diabetes Spectr. 2017;30(2):124–36. 10.2337/ds16-0004. 28588379 10.2337/ds16-0004 PMC5439356

[35] Wu C, Wu Z, Yang L, Zhu W, Zhang M, Zhu Q, et al. Evaluation of the clinical outcomes of telehealth for managing diabetes: A PRISMA-compliant meta-analysis. Medicine (Baltimore). 2018;97(43):e12962. 10.1097/MD.0000000000012962. 30412116 10.1097/MD.0000000000012962 PMC6221638

[36] van den Berg N, Schumann M, Kraft K, Hoffmann W. Telemedicine and telecare for older patients–a systematic review. Maturitas. 2012;73(2):94–114. 10.1016/j.maturitas.2012.06.010. 22809497 10.1016/j.maturitas.2012.06.010

[37] Marcolino MS, Maia JX, Alkmim MB, Boersma E, Ribeiro AL. Telemedicine application in the care of diabetes patients: systematic review and meta-analysis. PLoS ONE. 2013;8(11):e79246. 10.1371/journal.pone.0079246. 24250826 10.1371/journal.pone.0079246 PMC3826722

[38] Greenwood DA, Young HM, Quinn CC. Telehealth Remote Monitoring Systematic Review: Structured Self-monitoring of Blood Glucose and Impact on A1C. J Diabetes Sci Technol. 2014;8(2):378–89. 10.1177/1932296813519311. 24876591 10.1177/1932296813519311 PMC4455426

[39] Jalil S, Myers T, Atkinson I. A meta-synthesis of behavioral outcomes from telemedicine clinical trials for type 2 diabetes and the Clinical User-Experience Evaluation (CUE). J Med Syst. 2015;39(3):28. 10.1007/s10916-015-0191-9. 25677954 10.1007/s10916-015-0191-9

[40] Mushcab H, Kernohan WG, Wallace J, Martin S. Web-Based Remote Monitoring Systems for Self-Managing Type 2 Diabetes: A Systematic Review. Diabetes Technol Ther. 2015;17(7):498–509. 10.1089/dia.2014.0296. 25830528 10.1089/dia.2014.0296

[41] Wickramasinghe SI, Caffery LJ, Bradford NK, Smith AC. Enablers and barriers in providing telediabetes services for Indigenous communities: A systematic review. J Telemed Telecare. 2016;22(8):465–71. 10.1177/1357633X16673267. 27799449 10.1177/1357633X16673267

[42] So CF, Chung JW. Telehealth for diabetes self-management in primary healthcare: A systematic review and meta-analysis. J Telemed Telecare. 2018;24(5):356–64. 10.1177/1357633X17700552. 28463033 10.1177/1357633X17700552

[43] Correia JC, Meraj H, Teoh SH, Waqas A, Ahmad M, Lapao LV, et al. Telemedicine to deliver diabetes care in low- and middle-income countries: a systematic review and meta-analysis. Bull World Health Organ. 2021;99(3):209–19. 10.2471/BLT.19.250068. 33716343 10.2471/BLT.19.250068 PMC7941107

[44] De Groot J, Wu D, Flynn D, Robertson D, Grant G, Sun J. Efficacy of telemedicine on glycaemic control in patients with type 2 diabetes: A meta-analysis. World J Diabetes. 2021;12(2):170–97. 10.4239/wjd.v12.i2.170. 33594336 10.4239/wjd.v12.i2.170 PMC7839169

[45] Hangaard S, Laursen SH, Andersen JD, Kronborg T, Vestergaard P, Hejlesen O, et al. The Effectiveness of Telemedicine Solutions for the Management of Type 2 Diabetes: A Systematic Review, Meta-Analysis, and Meta-Regression. J Diabetes Sci Technol. 2021;17(3):794–825. 10.1177/19322968211064633. 34957864 10.1177/19322968211064633 PMC10210100

[46] McDaniel CC, Kavookjian J, Whitley HP. Telehealth delivery of motivational interviewing for diabetes management: A systematic review of randomized controlled trials. Patient Educ Couns. 2021;105(4):805–20. 10.1016/j.pec.2021.07.036. 34366228 10.1016/j.pec.2021.07.036 PMC8912910

[47] Anderson A, O’Connell SS, Thomas C, Chimmanamada R. Telehealth Interventions to Improve Diabetes Management Among Black and Hispanic Patients: a Systematic Review and Meta-Analysis. J Racial Ethn Health Disparities. 2022;9(6):2375–86. 10.1007/s40615-021-01174-6. 35000144 10.1007/s40615-021-01174-6 PMC8742712

[48] Robson N, Hosseinzadeh H. Impact of Telehealth Care among Adults Living with Type 2 Diabetes in Primary Care: A Systematic Review and Meta-Analysis of Randomised Controlled Trials. Int J Environ Res Public Health. 2021;18(22):12171. 10.3390/ijerph182212171. 34831925 10.3390/ijerph182212171 PMC8622760

[49] Sim R, Lee SWH. Patient Preference and Satisfaction with the Use of Telemedicine for Glycemic Control in Patients with Type 2 Diabetes: A Review. Patient Prefer Adherence. 2021;15:283–98. 10.2147/PPA.S271449. 33603347 10.2147/PPA.S271449 PMC7882448

[50] Rouleau G, Gagnon M-P, Côté J, Payne-Gagnon J, Hudson E, Dubois C-A. Impact of Information and Communication Technologies on Nursing Care: Results of an Overview of Systematic Reviews. J Med Internet Res. 2017;19(4):e122. 10.2196/jmir.6686. 28442454 10.2196/jmir.6686 PMC5424122

[51] Goharinejad S, Hajesmaeel-Gohari S, Jannati N, Goharinejad S, Bahaadinbeigy K. Review of Systematic Reviews in the Field of Telemedicine. Med J Islam Repub Iran. 2021;35:184. 10.47176/mjiri.35.184. 36042824 10.47176/mjiri.35.184 PMC9391764

[52] Lee PA, Greenfield G, Pappas Y. The impact of telehealth remote patient monitoring on glycemic control in type 2 diabetes: a systematic review and meta-analysis of systematic reviews of randomised controlled trials. BMC Health Serv Res. 2018;18(1):495. 10.1186/s12913-018-3274-8. 29940936 10.1186/s12913-018-3274-8 PMC6019730

[53] Timpel P, Oswald S, Schwarz PEH, Harst L. Mapping the Evidence on the Effectiveness of Telemedicine Interventions in Diabetes, Dyslipidemia, and Hypertension: An Umbrella Review of Systematic Reviews and Meta-Analyses. J Med Internet Res. 2020;22(3):e16791. 10.2196/16791. 32186516 10.2196/16791 PMC7113804

[54] Dixon RF, Zisser H, Layne JE, Barleen NA, Miller DP, Moloney DP, et al. A Virtual Type 2 Diabetes Clinic Using Continuous Glucose Monitoring and Endocrinology Visits. J Diabetes Sci Technol. 2020;14(5):908–11. 10.1177/1932296819888662. 31762302 10.1177/1932296819888662 PMC7477772

[55] Shaw S, Wherton J, Vijayaraghavan S, Morris J, Bhattacharya S, Hanson P, et al. Advantages and limitations of virtual online consultations in a NHS acute trust: the VOCAL mixed-methods study. Health Services and Delivery Research. 2018;6(21). 10.3310/hsdr06210.11/07/2024. 29939519

[56] Saeed SA, Masters RM. Disparities in Health Care and the Digital Divide. Curr Psychiatry Rep. 2021;23(9):61. 10.1007/s11920-021-01274-4. 34297202 10.1007/s11920-021-01274-4 PMC8300069

