# Supplementary material for: Effect of virtual care in type 2 diabetes management – a systematic umbrella review of systematic reviews and meta-analysis
Source: BMC Health Serv Res. 2025 Mar 6;25:348. doi: 10.1186/s12913-025-12496-0 (PMC11884068; doi:10.1186/s12913-025-12496-0)
Supplement: Supplementary file 1 — Supplementary Material 1. [file 12913_2025_12496_MOESM1_ESM.pdf]

S1: Search strategy for each database

	Databases	Search strategy (filtered for publications from 2011 to 2022)	Results (no of studies)	Date
1.	PubMed	(diabetes[Title/Abstract]) AND ((virtual[Title/Abstract]) OR (telehealth[Title/Abstract]) OR (telemedicine[Title/Abstract]))	2189	14/02/2022
2.	Embase	(diabetes and (virtual or telehealth or telemedicine)).tw.	3117	18/02/2022
3.	Medline	(diabetes and (virtual or telehealth or telemedicine)).tw.<br>Limit 2011-2017 (734); 2018 – 2020 (579); 2021 -current (491)	1804	18/02/2022
4.	CINAHL	((TI diabetes OR AB diabetes)) AND (((TI virtual OR AB virtual)) OR ((TI telehealth OR AB telehealth)) OR ((TI telemedicine OR AB telemedicine)))<br>Limit to 2011 to 2022	1061	21/02/2022
5.	Scopus	TITLE-ABS (diabetes) AND (TITLE-ABS (virtual) OR TITLE-ABS (telehealth) OR TITLE-ABS (telemedicine)) + PUB YEAR AFT 2010<br>Excluded note, letter, editorial, book, erratum, book chapter, conference paper, conference review	1930	03/03/2022
6.	Cochrane CENTRAL	(diabetes) ti OR (diabetes) ab; (virtual or telehealth or telemedicine) ti OR (virtual or telehealth or telemedicine) ab;	607	03/03/2022
		**Total studies**	**10,708**	

1. PubMED

Enter the search string in the search bar:

```
(diabetes[Title/Abstract]) AND ((virtual[Title/Abstract]) OR
(telehealth[Title/Abstract]) OR (telemedicine[Title/Abstract]))
```

Filter applied for publications from 1 Jan 2011 to the search date

Results: 2189 articles

2. Embase

Enter the search string in the search bar and apply publication year limit:

```
(diabetes and (virtual or telehealth or telemedicine)).tw.
limit 1 to yr="2011 -Current"
```

Results: 3117

3. Medline

Enter the search string in the search bar and apply publication year limits:

```
(diabetes and (virtual or telehealth or telemedicine)).tw.
limit 1 to yr="2011 -Current"                                    1804
(diabetes and (virtual or telehealth or telemedicine)).tw.
limit 3 to yr="2011 - 2017"                                      734
(diabetes and (virtual or telehealth or telemedicine)).tw.
limit 9 to yr="2018 - 2020"                                      579
(diabetes and (virtual or telehealth or telemedicine)).tw.
limit 11 to yr="2021 -Current"                                   491
```

Results: 1804

4. CINAHL
Enter the search string in search bar:

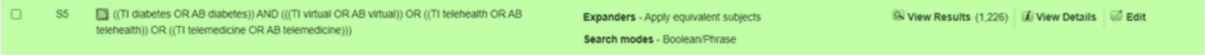

Limit the publications from Jan 2011 to current:

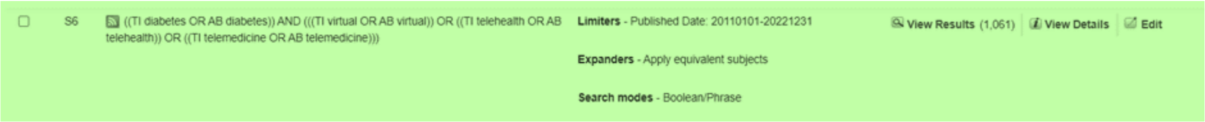

Results: 1061

5. Scopus

Enter the search string in search bar:

TITLE-ABS (diabetes) AND (TITLE-ABS (virtual) OR TITLE-ABS (telehealth) OR TITLE-ABS (telemedicine))
apply the filter for year range from 2011 onwards
Apply document type filter to exclude: conference paper, book chapter, conference review, note, letter, editorial, erratum, book

TITLE-ABS (diabetes) AND (TITLE-ABS (virtual) OR TITLE-ABS (telehealth) OR TITLE-ABS (telemedicine)) AND PUBYEAR > 2010 AND PUBYEAR < 2023 AND (EXCLUDE (DOCTYPE , "cp") OR EXCLUDE (DOCTYPE , "ch") OR EXCLUDE (DOCTYPE , "cr") OR EXCLUDE (DOCTYPE , "no") OR EXCLUDE (DOCTYPE , "le") OR EXCLUDE (DOCTYPE , "ed") OR EXCLUDE (DOCTYPE , "er") OR EXCLUDE (DOCTYPE , "bk"))

Results: 1930

6. Cochrane Central

#1 diabetes.ti. or diabetes.ab.
#2 (virtual or telehealth or telemedicine).ti. or (virtual or telehealth or telemedicine).ab.
#3 (#1) and (#2)
limit #3 to yr="2011 -Current"

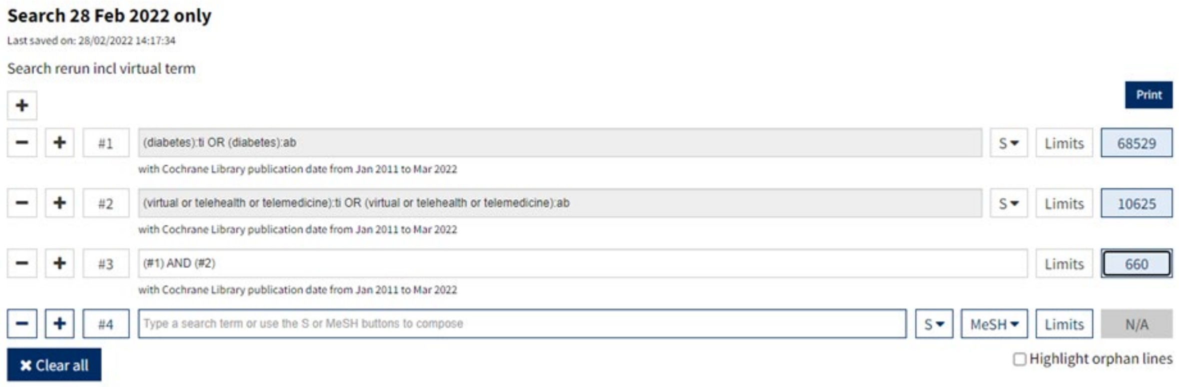

Hits 660 articles that included 8 reviews and 648 trials. But it showed publications from 2000 onwards because those were added to Cochrane centre after 2011.
When filter applied for year first published from 2011 to 2022, number of trials went down to 599.

Results: 607
