# Supplementary figures and images for: Effect of virtual care in type 2 diabetes management – a systematic umbrella review of systematic reviews and meta-analysis
Source: BMC Health Serv Res. 2025 Mar 6;25:348. doi: 10.1186/s12913-025-12496-0 (PMC11884068; doi:10.1186/s12913-025-12496-0)

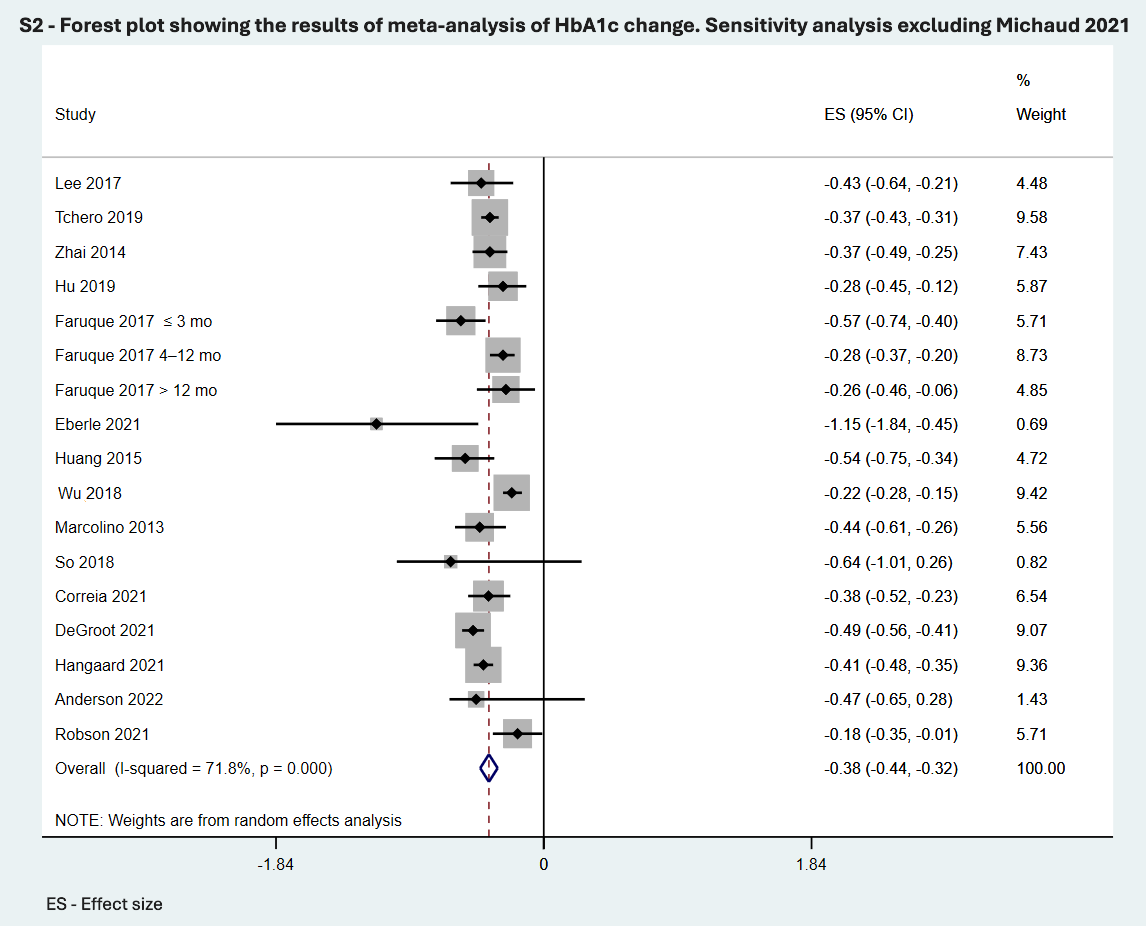

Supplement: Supplementary file 2 — Supplementary Material 2. [file 12913_2025_12496_MOESM2_ESM.png]
