# Supplementary material for: Effect of virtual care in type 2 diabetes management – a systematic umbrella review of systematic reviews and meta-analysis
Source: BMC Health Serv Res. 2025 Mar 6;25:348. doi: 10.1186/s12913-025-12496-0 (PMC11884068; doi:10.1186/s12913-025-12496-0)
Supplement: Supplementary file 3 — Supplementary Material 3. [file 12913_2025_12496_MOESM3_ESM.pdf]

S3: Quality assessment results using Joanna Briggs Institute critical appraisal checklist for systematic reviews an evidence synthesis

Study	Q1	Q2	Q3	Q4	Q5	Q6	Q7	Q8	Q9	Q10	Q11	Yes %	Overall quality
**Anderson 2022**	Y	Y	Y	Y	Y	Y	Y	Y	Y	Y	Y	100	H
**Cassimatis 2012**	N	Y	U	Y	Y	U	U	Y	N	U	Y	45	L
**Cong 2018**	Y	Y	Y	Y	Y	Y	Y	N	N	Y	Y	82	H
**Correia 2021**	Y	Y	Y	Y	Y	Y	Y	Y	Y	Y	Y	100	H
**DeGroot 2021**	Y	Y	Y	Y	Y	N	Y	Y	Y	Y	Y	91	H
**Eberle 2021**	U	N	Y	Y	U	U	U	U	Y	Y	U	36	L
**Faruque 2017**	Y	Y	Y	Y	Y	Y	Y	Y	Y	Y	Y	100	H
**Greenwood 2014**	Y	Y	Y	Y	N	N	N	N/A	N	Y	Y	55	M
**Hangaard 2021**	Y	Y	U	Y	Y	Y	Y	Y	Y	Y	N	82	H
**Hossain 2019**	N	U	Y	Y	Y	Y	Y	N/A	N/A	Y	Y	64	M
**Hu 2019**	Y	Y	Y	Y	Y	Y	Y	U	Y	Y	Y	91	H
**Huang 2015**	Y	Y	Y	Y	N	Y	Y	Y	Y	Y	Y	91	H
**Jalil 2015**	Y	Y	Y	Y	U	U	U	Y	N	Y	Y	64	M
**Kaveh 2021**	Y	Y	U	Y	Y	U	U	N/A	N/A	U	U	36	L
**Lee 2017**	U	Y	Y	Y	Y	N	Y	Y	Y	Y	Y	82	H
**Marcolino 2013**	Y	Y	Y	Y	Y	N	Y	Y	Y	Y	Y	91	H
**Marsh 2021**	Y	Y	Y	Y	Y	N	N	N	N	U	U	45	L
**McDaniel 2021**	Y	Y	Y	Y	Y	Y	U	N	N	Y	Y	73	H
**McLendon 2017**	Y	Y	Y	U	U	U	U	N/A	N/A	Y	Y	45	L
**Michaud 2021**	Y	Y	Y	Y	N	Y	Y	Y	Y	Y	Y	91	H
**Mushcab 2015**	Y	Y	Y	Y	N	N	N	N/A	N/A	U	Y	45	L
**Robson 2021**	Y	Y	Y	Y	Y	N	N	Y	N	Y	Y	73	H
**Sim 2021**	Y	N	Y	Y	N/A	N/A	Y	N/A	N/A	Y	Y	55	M
**So 2018**	Y	Y	Y	Y	Y	Y	U	Y	N	Y	Y	82	H
**Su 2016**	Y	Y	Y	Y	N	N	Y	Y	Y	Y	Y	82	H
**Su 2016**	Y	Y	U	Y	N	N	Y	Y	Y	Y	Y	73	H
**Tchero 2019**	Y	Y	Y	U	Y	N	Y	Y	Y	U	U	64	M
**VanDenBerg 2012**	Y	Y	Y	Y	N	N	U	N/A	N	U	U	36	L
**Wickramasinghe 2016**	Y	Y	Y	Y	Y	Y	Y	N/A	N/A	Y	Y	82	H
**Zhai 2014**	Y	Y	Y	Y	Y	Y	Y	Y	Y	Y	Y	100	H

Extracted papers were considered "low quality" if the results were < 50%, "moderate quality" if they fell between 50 and 69%, and paper(s) that received > 69% were considered "high quality"

Q1: Is the review question clearly and explicitly stated?
Q2: Were the inclusion criteria appropriate for the review question?
Q3: Was the search strategy appropriate?
Q4: Were the sources and resources used to search for studies adequate?
Q5: Were the criteria for appraising studies appropriate?
Q6: Was critical appraisal conducted by two or more reviewers independently?
Q7: Were there methods to minimise errors in data extraction?
Q8: Were the methods used to combine studies appropriate?
Q9: Was the likelihood of publication bias assessed?
Q10: Were recommendations for policy and/or practice supported by the reported data?
Q11: Were the specific directives for new research appropriate?

Y Yes, N No, U Unclear, N/A Nonapplicable, L Low, M Moderate, H High
